# Perspectives and Needs of Social Support for People With HIV: A Qualitative Study According to Three Aspects of Stakeholders

**DOI:** 10.1155/nrp/8847734

**Published:** 2025-10-27

**Authors:** Bo Zhou, Dongmei Li, Lijian Huang, Shuyu Han, Lili Zhang, Xiulian Wu, Dongxia Wu, Keyi Chang

**Affiliations:** ^1^School of Nursing, Peking University, Beijing 100191, China; ^2^Department of Infection and Immunology, Beijing Youan Hospital Affiliated with Capital Medical University, Beijing 100069, China

**Keywords:** AIDS, HIV, qualitative research, social support

## Abstract

**Aims:**

To explore the perspectives and needs of people with HIV in terms of social support from the three perspectives of healthcare professionals, peer volunteers, and people with HIV.

**Background:**

Progress in ART has pushed HIV treatment to improve the quality of life of infected people, and UNAIDS has promoted the “95” goal, emphasizing good quality of health. Social support is critical to HIV health management and is strongly associated with multiple health outcomes. The current research on social support in HIV field is mostly based on traditional classification and requires deeper exploration of multidimensional influences.

**Methods:**

During the period from January to March 2024, face-to-face, semistructured, in-depth interviews were conducted at Beijing You'an Hospital. The data were analyzed using an inductive thematic analysis.

**Results:**

A total of six people with HIV, three peer volunteers, and seven medical professionals were interviewed. Inductive analysis of the purposive samples revealed that the social support needs of people with HIV encompass five dimensions: informational support, instrumental support, emotional support, affiliational social support, and appraisal social support.

**Conclusions:**

The social support required by people with HIV is multidimensional in nature. In clinical practice, it is essential to pay full attention to the individual characteristics of people with HIV and develop personalized support plans to ensure that people with HIV receive comprehensive social support.

## 1. Introduction

With the rapid development of antiretroviral therapy (ART), HIV infection has become a chronic condition [1]. The destination of AIDS treatment is no longer viral suppression, but to improve the healthy quality of life of people with HIV (PWH) [2]. The UNAIDS has also proposed the fourth “95” to help PWH, which means that 95% of PWH who have been treated with ART have a good healthy quality of life [3]. However, to achieve the fourth “95” will encounter many barriers, for instance, physical problems, behavioral problems, psychological problems, and social discrimination. Therefore, the UNAIDS also calls for more funding for PWH treatment and care to promote more resources and help for PWH.

In addition to the professional medical resources provided by hospitals and institutions, social support is essential for the health management of PWH. Previous literature has shown that social support is significantly associated with many health outcomes of PWH, including depression, stigma, discrimination, viral suppression, and medication adherence [4–8]. At present, most quantitative studies related to social support in the field of HIV are based on the classical concept of social support. For example, Xiao divided social support into two categories; one is subjective, and the other is objective [9]. House et al. divide social support into instrumental support and emotional support; Barrera divides social support into three categories: embeddedness, perceived social support and enacted support [10–12]. However, in the ART era, most PWH face increasingly complex issues such as social and cultural development not keeping up with medical development, and PWH will suffer more discrimination. The life expectancy of PWH continues to increase, yet PWH still face prevalent and severe discrimination [13]. These simple dimensions of social support are clearly no longer applicable to PWH. The classical concept of social support may not fully explain the dimensions of PWH social support needs. In order to ensure the long-term well-being of PWH, it is necessary for us to clarify the specific categories of social support needed by them in this new age. Therefore, it is necessary to understand the comprehensive social support needs of PWH beyond the previous concept of social support.

To provide safer and more precise social support for PWH, it is paramount to understand their genuine needs. However, the stigma and shame associated with the disease often discourage PWH from disclosing their status and discussing disease-related information, posing challenges to our research endeavors [14]. Amidst the complexities of HIV health management and the widespread prevalence of HIV-related discrimination, medical professionals involved in HIV treatment and care as well as HIV peer volunteers has emerged as vital sources of social support for PWH. Their experiences enabled them to penetrate barriers and comprehend the needs of PWH. Therefore, our study conducted a qualitative examination of HIV-related social support needs from the three perspectives of medical professionals, peer volunteers, and PWH, aiming to offer fresh insights and references for future research and practices in HIV-related social support.

## 2. Methods

### 2.1. Design

The present study is a qualitative and descriptive study to explore in depth the perspectives and needs of PWH in terms of social support from the tripartite perspectives of medical professionals, peer volunteers, and PWH through purposive sampling and one-on-one semistructured in-depth interviews. This research was conducted according to the guidelines in the Standards for Reporting Qualitative Research (SRQR) checklist.

### 2.2. Setting and Participants

The study was conducted at Beijing You'an Hospital from January to March 2024, targeting medical professionals specializing in AIDS care, PWH undergoing routine follow-ups, and peer volunteers from Love Home as participants for interviews. The inclusion criteria for PWH were ① aged over 18 years; ② having initiated ART; ③ willingness to participate voluntarily and ability to complete the study. For medical professionals, the inclusion criteria were ① employment in the Department of Infectious Diseases and Immunology; ② more than 5 years of fixed-position work experience; ③ holding a bachelor's degree or above. As for peer volunteers, the inclusion criteria were as follows: ① confirmed diagnosis of HIV infection; ② possessing more than 3 years of experience in peer support work.

### 2.3. Data Collection

This study used a semistructured, in-depth interview method to collect data. Before the interviews, the researchers fully familiarized themselves with the content of the interview outline and mastered the relevant interview method techniques. They fully informed the participants of the purpose, significance, and confidentiality of the study and obtained informed consent from the participants by signing an informed consent form. The interviews ensured a quiet and comfortable environment to protect the privacy of participants. Each interview lasted approximately 40–60 min. The entire interview was audio-recorded with the participant's consent, and the participants' body movements and expressive nonverbal behaviors were recorded. The sample size for this study was obtained until the interviews reached information saturation.

We inquired with PWH regarding their experiences of receiving social support following diagnosis, their perspectives on the support needs of PWH, and their views on who would be best suited to offer various types of support. Medical professionals were interviewed about their experiences in supporting PWH, their evaluation of the support needs of PWH, and their opinions on suitable support providers. Peer volunteers shared their motivations for engaging in volunteer work, their experiences in providing support to PWH, and their insights into the support needs of PWH and the most appropriate support providers.

### 2.4. Data Analysis

Two researchers employed NVIVO software (version 11.0) to transcribe the audio recordings of the interviews within a 24-h timeframe. Subsequently, they organized and synthesized the data using a rigorous thematic analysis approach. This involved extracting core ideas from the vast array of linguistic descriptions and coding, subsequently consolidating recurring concepts into coherent themes. This process aimed to ensure academic rigor and precision in the data interpretation.

## 3. Results

A total of 6 PWH (aged 25–49 years, median age 33.5 years, infected with HIV for 0.5–16 years, including 3 with bachelor's degrees, 2 with high school diplomas, and 1 with a junior high school diploma; hereafter referred to as “P”), 3 peer volunteers (aged 35–41 years, median age 41 years, infected with HIV for 8–20 years, including 1 with a bachelor's degree and 2 with secondary vocational school diplomas; hereafter referred to as “PV”) and 7 medical professionals (aged 28–46 years, median age 37 years, including 6 with bachelor's degrees and 1 with a master's degree; hereafter referred to as “MP”) were included in the study.

**Figure 1:**
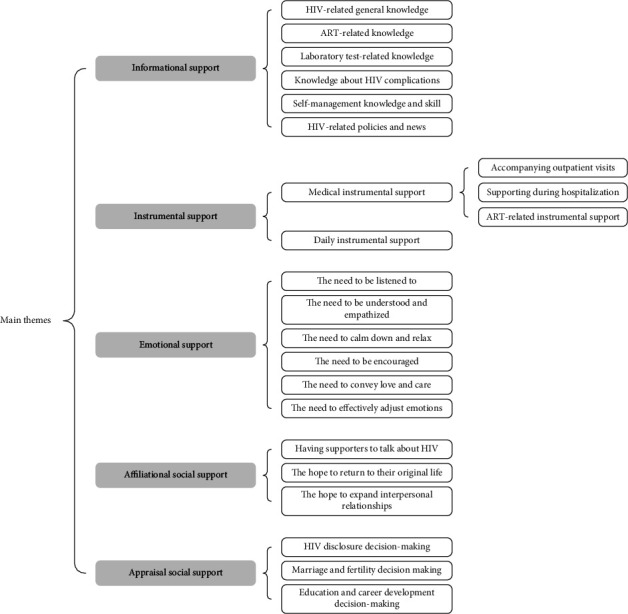
Summary of main themes and subthemes.

As shown in Figure 1, the study identified five main themes related to social support for PWH: informational support, instrumental support, emotional support, affiliational social support, and appraisal social support.

### 3.1. Theme: Informational Support

#### 3.1.1. HIV-Related General Knowledge

After being diagnosed with HIV, PWH have a strong need to understand HIV-related basic knowledge.

“One of the focuses of our work is to publicize the basic knowledge related to HIV and how to seek medical treatment and effective treatment.” (PV3)

“When I learned that I was infected with HIV, the first thing I wanted to know was how long I could live, and I was quite scared at that time, but later on, through the volunteers explaining HIV-related knowledge to me, I gradually became less nervous and fearful.” (P2)

#### 3.1.2. ART-Related Knowledge

Many PWH said that ART is the information they are most concerned about. They are eager to confirm whether their treatment is effective to learn about the progress of ART.

“Initially, their biggest concern is about the treatment aspect, and after the medication is administered, we take the initiative to educate about compliance and behavioral education.” (PV1)

“Before taking the medication, they have a lot of concerns because they lack knowledge in this area. We inform people about the cost of the medication, the benefits of taking the medication, the side effects of the medication, and the precautions to be taken.” (PV2)

“The main concern is the new development of antiviral treatment, which I am personally more concerned about, as well as the adherence to the medication and the side effects of each medication.” (P1)

“I hope the volunteers can organize more lectures or exchanges on drugs, treatment, and academics.” (P3)

#### 3.1.3. Laboratory Test-Related Knowledge

Laboratory test-related knowledge is also a major concern for PWH. After each follow-up visit, they hoped that someone would help confirm whether there were any problems with the test reports.

“Every time the test results come out, they will receive them on their cell phone and they will take a screenshot and ask us to interpret it for them.” (PV1)

“PWH don't understand the meaning of this lab indicator and usually consult us after the results come out.” (PV2)

“If I find any abnormality in the labs, I will contact the volunteer and he will help me answer the question, if he can't answer the question, he will ask the doctor for me and tell me the result.” (P1)

“When I find an abnormality in the indicator, I will send it to my mom and ask her what is going on.” (P2)

“After every review my labs will ask the volunteer to help me see if there are any problems, I can't read them myself.” (P4)

#### 3.1.4. Knowledge About HIV Complications

PWH hope to learn about HIV complication-related knowledge to help them identify and treat related diseases early.

“Some of them will tweet us for help if they are found and not treated in time, they will develop some opportunistic infections such as pneumonia and tuberculosis.” (PV2)

“We will tell them to take their medication properly or else they will develop opportunistic infections if the treatment is not effective, and we will also tell them what opportunistic infections they may have in case they develop any symptoms so that they can seek medical attention in time.” (MP3)

“I am worried about the development of drug resistance during the process of taking medication so I will ask the volunteers about opportunistic infections so that I can detect and treat any complications in time.” (P5)

#### 3.1.5. Self-Management Knowledge and Skill

PWH also pay attention to daily self-management, which includes taking supplements to improve immunity, maintaining a healthy body, and managing side effects of medications.

“In addition to knowledge about antiretroviral treatment, the next thing they wanted to know was about daily nutrition and health care, including work and rest schedules, how to consult a doctor if they were not feeling well, and whether there was a conflict between other chronic disease medications and HIV medications.” (MP7)

“For example, what should I pay attention to in terms of life, what nutrition should be increased for HIV-infected people, including contraindications to eating during the course of taking medication.” (P2)

“I am more concerned about my personal health, one kind of daily health management and one kind of side effect aspect of the medication.” (P4)

“I don't know what I should pay attention to in my life after infection, and these friends of mine also give me some self-management advice, such as taking some Vitamin C and so on.” (P3)

#### 3.1.6. HIV-Related Policies and News

PLWH are also interested in HIV-related policies and news because they may be closely related to their own rights and interests.

“They will inquire about what kind of legal responsibility they have to bear if they infect each other, and we will also take the initiative to inform him about such questions when they are first found to be positive.” (MP4)

“Some of our friends will be concerned about this knowledge, and we will explain it to them, because our country has regulations on HIV prevention and control, as well as the law on infectious diseases, and our rights are protected by the law.” (PV2)

“He may ask what good policies the country's four exemptions and one care, and what good policies the treatment can enjoy, and we will give him certain help and guidance for his actual situation.” (MP6)

“What good policies can the country give us, this is also my more off and key question.” (P1)

“I am most concerned about the latest news related to HIV, such as what new drugs have appeared, and Chinese medicine treatment for HIV.” (P5)

### 3.2. Theme: Instrumental Support

#### 3.2.1. Medical Instrumental Support

##### 3.2.1.1. Accompanying Outpatient Visits

Unfamiliarity with the environment and procedures, along with the stigma of HIV, makes many PWH reluctant to go to the clinic alone, preferring to have someone accompany them during their visits.

“For the follow-up visit, for those who come for the first time, we can accompany them on a one-to-one basis, and some of them may be less capable on their own, so they have to find volunteers to accompany them every time they come for follow-up visits.” (MP1)

“Every time they come to the hospital, they are not accompanied by their family members, and they come alone more often, so they usually ask us to accompany them to the outpatient clinic.” (PV3)

“If I am not feeling well I will come to the hospital to see the volunteers and ask them to take me to the doctor.” (P1)

“The volunteers told me that if you come to the hospital and don't have anyone with you or if you don't know what to do, I will come and help you and accompany you to the outpatient clinic. This is very touching to me because sometimes I feel lonely if I go to the hospital by myself.” (P4)

“I needed someone to accompany me at the earliest stage, I didn't know anything at the early stage, I was in the hospital like a fly on the wall, I still needed them to guide me.” (P6)

##### 3.2.1.2. Supporting During Hospitalization

Many PWH choose not to inform their families about their HIV status, leading to concerns about having no one to care for them if needed during hospitalization.

“If it involves hospitalization or requires a family member's signature, I may have to ask for volunteers to help, and I don't dare to ask for help with this illness.” (P1)

“Once, I had acute gastroenteritis and my family wasn't around, luckily a pretty good friend, who took time off work to take me to the doctor, took care of me in the hospital.” (P3)

“If one day I am hospitalized for this or any other illness and don't have my family around, I hope they can help me.” (P5)

“Those who are sicker on the ward will have to tell their families because they need the support and care of others.” (MP2)

“The vast majority of PWH who are hospitalized with opportunistic infections will have told their family because they need to be taken care of, but this is a passive way of telling the family, there's no way around it.” (MP3)

##### 3.2.1.3. ART-Related Instrumental Support

PWH hope to obtain timely help to maintain medication adherence when they are out of medication due to various emergencies.

“Like during the holiday season …um…forgot my medication, and then can I borrow a medication ah or coordinate it ah.” (PV3)

“When I was working out of town, it was the volunteers who mailed my medication to make sure I didn't stop taking it.” (P1)

“I was temporarily living out of town during that time, and it was too time-consuming to travel a long distance to pick up my medication every time I came for a medical checkup, so I asked them to send it to me when I didn't need a medical checkup.” (P2)

“It's difficult to take time off from work in our organization, so I asked the volunteers to mail it to me when I didn't need a medical checkup, which was much more convenient.” (P4)

“They mailed us medication during the epidemic, so that was good, and it's still very thoughtful to provide a service to mail medication if something prevents you from receiving it in time.” (P6)

“Many patients need us to break down the ‘how'. How to use a pill box, how to order refills on time, how to take them with food… that hands-on guidance is crucial.” (MP7)

#### 3.2.2. Daily Instrumental Support

The participants indicated that some PWH were not willing to inform their families when they faced difficulties in their lives and hoped to receive help from others.

“If they encounter difficulties in their lives, fewer of them seek help directly from their family members, and most of them find other excuses to seek help from their relatives and friends, probably for fear that their family members will know about it. We can apply for some special hardship funds every year, but this fund is also limited.” (MP4)

“It is determined that the financial condition is not good, we can help him to apply for some relief, 6000 dollars for opportunistic infections relief, that is to help him as much as possible.” (MP5)

“Because there are really a lot of people who, due to the cost, have no choice but to go for free medication, or due to financial problems, he doesn't even take the medication, and for them they need to be provided with help in their lives.” (P3)

### 3.3. Theme: Emotional Support

#### 3.3.1. The Need to Be Listened to

Participants mentioned that talking helps relieve stress and provides sound advice, and they expect to be listened to.

“They need to be listened to a lot, it relieves stress, but be careful not to tell it to just anyone, make sure that person understands you, accepts you and helps you, otherwise don't say anything if it's counterproductive.” (PV3)

“There are times when I would like to go and talk to someone about the infection, but I can't say it yet, if I have a few people I can talk to, then surely I feel differently in my heart.” (P1)

“After I confide in them, they give me advice, but my focus is not on listening to his advice, it's more important because I have someone to confide in.” (P3)

#### 3.3.2. The Need to Be Understood and Empathized

PWH expressed hope that someone would understand and empathize with them.

“When the mood is depressing, they talk to the volunteers because they are all infected and can empathize.” (MP3)

“People mainly need psychological support. Because this problem is not like other problems, everyone can talk about it, it is still difficult to talk about it.” (PV2)

“They are very eager to have someone to understand his difficulties, but then they may feel that HIV infection is a dirty disease, and there is a certain sense of shame, and they don't want to tell people about it and feel ashamed.” (MP7)

“For example, I once told a good friend about the infection and he ended up crying, I was happy to be understood by him.” (P1)

“My sister cares more about me, this is something she never asks me about, she says you just have a happy life, I am touched to be understood by her.” (P6)

#### 3.3.3. The Need to Calm Down and Relax

Participants expressed a desire for people or environments that helped them relax and minimize the distress caused by stress.

“A lot of infected people who come to do errands near the hospital and pass by come and chat about some recent unpleasantness. They can relax because of our environment.” (MP1)

“When he passes by the hospital he may ask me where are you? Looking for me by the way to have a chat, because they are here, they will be very relaxed facing us, and we will also ask how have you been? I have pill boxes here, would you like to take one?” (MP5)

“If I have any questions, I ask the volunteers. I don't talk to my colleagues or family members for fear of being found out by others, but I feel very relaxed in the volunteers' place.” (P1)

“I want to talk to others after I am infected, but who do I talk to? Actually I was very stressed and nervous.” (P5)

#### 3.3.4. The Need to Be Encouraged

Participants expressed that receiving encouragement would boost their confidence in managing the disease.

“He himself had a low immunity and lost confidence in the future. After our encouragement, his body recovered after six months of treatment, and he is now able to work normally, and is completely back to his normal state.” (MP2)

“If I meet a depressed person, I will tell him that I have been taking medication for 10 years, and he will look at me with a very surprised look, which is actually a kind of invisible encouragement and support.” (PV3)

“Although I need their support and encouragement, this certainly cannot tell them. Telling them would only make it worse, and it would be a home wrecker if I didn't.” (P5)

#### 3.3.5. The Need to Convey Love and Care

Participants indicated that infected individuals want to be loved and cared for by others and accepted by society.

“Volunteers, doctors and nurses also talk to me about how have you been? How's work going? Concerned about my living situation, they are all especially nice.” (P1)

“He said that healthcare workers and volunteers in Beijing give more care to infected people than here, and that I could talk to the volunteers about anything and they could help me, so I transferred to Beijing.” (P4)

“He advised me to take my medication and not to be sad, said a lot of caring words, told me not to think too much and said that if I was unhappy, I should come to him, which I felt quite warm.” (P5)

“That is my good friend whom I have known for twenty years, he will care about me a lot, I still feel quite warm.” (P6)

#### 3.3.6. The Need to Effectively Adjust Emotions

PWH expressed they need support to help them effectively adjust emotions.

“If they are psychologically stressed, most of them will go through outside sources, such as looking for HIV-friends or peer education for counseling, and adjusting the emotional stress brought about by stress through our guidance.” (MP5)

“They don't dare to tell others when they are emotionally unstable, in fact, they long for someone to enlighten them, but for the sake of privacy, most of them still have to adjust their responses by themselves.” (MP7)

“The head nurse shares some things with me, and with them, I feel much better after a long time, and usually when I participate in some activities organized over here and learn some knowledge my mood is not so bad.” (P1)

“I am sometimes unhappy, I usually talk to my HIV-friends, then we will sing, play ball games and sports together, and after that my mood will be better.” (P2)

### 3.4. Theme: Affiliational Social Support

#### 3.4.1. Having Supporters to Talk About HIV

Participants expressed that they needed supporters to be able to safely talk about HIV. This can help them build a sense of belonging and identity.

“People who are infected are usually in a lot of groups, because in this kind of group of infected people, they can talk about things about HIV without worrying, such as how to get medication, treatment or other problems.” (MP4)

“It is impossible to go to other hospitals with HIV problems, and at the same time, it is even more afraid to go to talk about it with the people around me, and I am sure that I am coming to a professional place, and luckily the staff are exceptionally nice and I shouldn't have to hide in the face of them.” (P1)

“I hope someone can accept me, but the fact is that if people find out that I have this disease, they will definitely stay away from me and think that I am not living a proper life, so I don't dare to talk to others.” (P4)

“I like to participate in the training activities organized by Caring Home, where I can communicate more deeply with senior volunteers and PWH.” (P6)

#### 3.4.2. The Hope to Return to Their Original Life

When HIV infection has a huge impact on PWH's life, they hope to get support to return to their original life as much as possible.

“For example, if he told his housemates, he might not be able to live together, if he told his colleagues, there is a possibility that he might not be able to keep his job. We will give them some advice to minimize the impact of AIDS on their working life.” (MP2)

“Give him some guidance on his life, such as psychological counseling, how to get along with his family, how to better integrate into society, etc.” (MP6)

“I think most of the infected people are economically disadvantaged because their jobs are not particularly stable, so their income is also relatively low, and regular medical checkups will have an impact on their finances and their lives, so we will also take the initiative to apply for a fee waiver for them, so that the disease will have less of an impact on their life.” (P1)

“At first I didn't know what to do subsequently, then I met someone online who shared with me the process of applying for antiviral drugs in Wuhan, and I was able to go through the treatment to return to my original life.” (P4)

“There are more friends who are positive after infection, they will often organize activities, and I also hope that the society will be more tolerant to us and can treat us equally to be able to live a normal life.” (P6)

#### 3.4.3. The Hope to Forge Meaningful Connections With Other PWH

The analysis revealed that while HIV infection often strained existing relationships, participants expressed a strong, specific desire to build new connections with peers who were also living with HIV.

“When PWH are together, they are more relaxed, they don't have to think so much about what they say, and they can get practical help, but with their classmates or family members, they are more sensitive. We often organize some training activities, and people are especially willing to participate because they want to meet more friends. We also have a WeChat group where people can usually go and chat about some of the problems they encounter.” (PV2)

“We can go hiking and play together, add each other's WeChat to expand our social circle, and get more help.” (PV3)

“I hope to participate in the activities to know more HIV-friends, to help each other with some problems that individuals do not understand.” (P3)

“I have to come to the hospital once every three months, and sometimes I also come when there are activities here because it is good to participate in the activities organized by them to get to know some friends.” (P4)

“I still have to go to the volunteers when I have a problem, and I hope to get to know more volunteers, who are still able to provide the most practical help.” (P6)

### 3.5. Theme: Appraisal Social Support

#### 3.5.1. HIV Disclosure Decision-Making

Affected by HIV-related discrimination, many PWH are troubled about whether to disclose their HIV status to their important social relationships and hope to get support to help them make decisions.

“For those who are already married, we will mobilize them to inform their spouses in the first place, while some will choose not to tell their spouses for fear that they will get divorced if they know about it, and we will mobilize them to bring their loved ones over for the test.” (MP2)

“There will be some people who have concerns about whether to tell their families, and we will weigh the pros and cons to help him analyze.” (MP5)

“For some common questions, such as whether to tell parents or friends after antiviral treatment, I will give him some advice on a case-by-case basis.” (MP7)

“After I was infected, the doctor told me not to tell anyone and not to tell my family, but after I thought about it, I really couldn't tell them either.” (P5)

#### 3.5.2. Marriage and Fertility Decision Making

Some PWH are confused about whether to get married and have children, and hope to get support to help them make decisions.

“For example, can I get married to a negative party if I am infected? We will give him the knowledge of mother-to-child transmission prevention.” (MP1)

“The more frequently asked questions are whether one wants to get married and whether having a child will result in the child being infected.” (MP3)

“For getting married, if you are sure about it, then you can choose to get married and also have healthy babies under scientific guidance and the other person will not be infected, which is definitely not a problem.” (MP6)

“I think getting married is still not acceptable to many people, so what to do in this case? I think we can only say that we should look for volunteers to find a doctor to help analyze and make a comprehensive judgment, and then make a decision in the light of our own actual situation.” (P1)

#### 3.5.3. Education and Career Development Decision-Making

When HIV infection affects PWH's important events such as education and career development, they hope to obtain support to help them make decisions.

“They themselves will have some anxiety ah, for example, those who are engaged in manual labor for a long time at work or those who work night shifts should they change their jobs?” (PV2)

“Because we have more contacts with people, we will help them to inquire about which workplaces do or don't test for HIV, and then we will tell them.” (PV3)

“I also asked the volunteers when I was choosing a job, and was told that only state-owned enterprises and those with staffing will test for HIV, and that ordinary training institutions will not test for HIV. If it was an ordinary training organization, they would not check this, so I chose to teach dance in the training organization.” (P4)

## 4. Discussion

We conducted a qualitative study examining the scope of social support among three groups: PWH, peer volunteers and medical professionals. Building on previously validated summaries, we further explored the social support needs of PWH and identified five distinct dimensions: informational, instrumental, emotional, affiliational, and appraisal support. This study not only enriches the theoretical understanding of social support in the context of HIV but also offers practical insights to inform the development of more comprehensive and individualized support strategies, thereby contributing to the advancement of social support research in this field.

Information support is critical for PWH due to multifaceted challenges arising from the nature of the disease and its social implications. HIV infection involves complex symptomatology, opportunistic infections, and frequent co-infections, complicating disease management and increasing health-related distress [15]. Lifelong ART, though effective, requires continuous education and personalized management due to its side effects and adherence demands. Furthermore, PWH face structural barriers such as financial hardship, employment discrimination, and HIV-related stigma, which impair social relationships and economic stability, thereby amplifying the need for reliable and multidimensional information [16]. Comprehensive information support, spanning medical, psychosocial, legal, and economic domains, is essential to facilitate informed decision-making and effective self-care [16–20]. Evidence supports the efficacy of digital health interventions in improving ART adherence and quality of life, such as SMS reminders and social media-based education [21, 22]. Thus, developing accessible, culturally adapted, and integrated information strategies remains imperative to support PWH in managing their health and social challenges.

Our findings indicate that PWH require emotional, instrumental, and affiliational social support. The necessity of emotional and instrumental support is well-established in the literature. Previous meta-analytic research has demonstrated that stigma negatively correlates with well-being and amplifies feelings of shame among PWH, underscoring the importance of psychosocial interventions to improve quality of life [23]. Similarly, longitudinal observational studies have reported that integrated support, including healthcare access and emotional care, can significantly enhance treatment adherence and promote self-worth among PWH [24]. In contrast, affiliational social support has received comparatively less attention. Nevertheless, given the pervasive social discrimination and isolation experienced by PWH, affiliational support is essential to facilitate healthy social relationships, mitigate disease-related challenges, and ultimately improve treatment outcomes and recovery.

Beyond the aforementioned support, providing appraisal support to PWH during critical life choices is crucial. HIV is often linked to social discrimination and AIDS-related stigma, which can be internalized by PWH, intensifying their unease, anxiety, and helplessness due to social rejection, thereby inducing social avoidance behaviors that impact their psychological state and decision-making [25]. The contagious and lifelong nature of HIV further complicates major life decisions, such as marriage, childbearing [26], and HIV status disclosure [27]. When PWH face decision-making hesitations and difficulties, we should respect their wishes, bolster their self-management confidence, establish efficient information exchange platforms or consultation channels, and offer tailored appraisal social support to enhance their quality of life and well-being, enabling them to better tackle disease-related challenges. A qualitative study on the decision-making trajectories and influencing factors of ART for PWH proposed that they undergo four phases: information gathering, decision designing, decision executing, and decision evaluation, influenced by decision-making motivation and resistance. Some PWH reported that short consultation times in healthcare services foster suspicion towards hospitals, hindering joint treatment decisions with doctors, whereas comprehensive social support can effectively alleviate negative emotions and boost confidence in overcoming the disease [28].

In clinical practice, it is essential to comprehensively address the diverse social support needs of PWH. Establishing a holistic support system requires multifaceted strategies to deliver comprehensive and individualized care. First, professional training for healthcare providers should be strengthened to enhance their expertise and competence in addressing the psychological, physiological, and social needs of PWH, enabling more precise and empathetic support. Second, public awareness and acceptance of HIV must be improved through health education to reduce stigma and discrimination, thereby fostering an inclusive environment that facilitates social integration and alleviates psychological burdens. Additionally, promoting peer support networks is critical; facilitating support groups can encourage emotional and informational exchange among PWH, which helps diminish isolation and strengthens mutual aid. It is equally important to account for variations in support needs across age, gender, and cultural backgrounds. Support strategies should be tailored to individual profiles and preferences, including the selection of suitable support providers. For example, newly diagnosed PWH require basic disease and treatment education, whereas long-term survivors may need emotional companionship and rehabilitation guidance. Special populations, such as pregnant women and children, need targeted interventions to prevent mother-to-child transmission and ensure early detection. Those experiencing socioeconomic hardship should receive necessary financial and living assistance. By integrating these personalized and multilevel approaches, we can more effectively meet the diverse needs of PWH, improve their quality of life, and promote broader public health and social harmony.

This study serves as a valuable reference for future research and practice pertaining to social support in the realm of AIDS; however, it is subject to several limitations. First, the sample population was confined solely to Beijing, thereby neglecting the potential impact of geographical specificity. Second, the interview participants were exclusively male, overlooking the potential disparities in social support experienced by female PWH compared to male PWH. Third, the study only included unmarried individuals, failing to account for the specific social support needs of PWH with marital and child-rearing backgrounds. Finally, the research process neglected to consider the unique social support requirements of PWH within the context of married life and child-rearing responsibilities.

## 5. Conclusion

In conclusion, this study underscores the critical importance of adopting a multidimensional approach to social support for PWH, encompassing not only material medical assistance, emotional support, and affiliational support, but also informational access, major decision-making assistance, and other nuanced needs. To accurately assess and address the diverse requirements of PWH, it is essential to develop comprehensive and specific assessment tools that can inform the design of personalized support strategies. Clinically, recognition of individual characteristics and the implementation of tailored support plans are paramount to delivering adaptable and holistic social support. By prioritizing both individualization and comprehensiveness in interventions, we can better respond to the evolving needs of PWH and ultimately enhance their overall well-being and quality of life.

## Data Availability

Due to the sensitive nature of the data and to ensure participant privacy, the raw data underlying this paper are not publicly shared. De-identified data can be made available by the corresponding author upon reasonable request and subject to the approval of the institutional ethics committee.
